# Polarization conversion from a thin cavity array in the microwave regime

**DOI:** 10.1038/srep09366

**Published:** 2015-03-23

**Authors:** B. Tremain, H. J. Rance, A. P. Hibbins, J. R. Sambles

**Affiliations:** 1 Electromagnetic and Acoustic Materials Group, Department of Physics and Astronomy, University of Exeter, Exeter. EX4 4QL, United Kingdom

## Abstract

Linearly polarized microwave radiation is shown to have its plane of polarization converted to the orthogonal state upon reflection from an ultrathin (*λ*/25) cavity array. The structure benefits from an uncomplicated design consisting of a metallic grating closely separated from a ground plane by a dielectric spacer. A single set of periodically spaced slits (monograting) exhibits polarization conversion when the normally incident electric field is aligned at 45° to the slits. Two orthogonal sets of slits (bigrating) allows this narrow-band effect to be broadened when the two orthogonal resonances are separated in frequency. We optimise the design and experimentally demonstrate near loss-less polarization conversion (95% of the incident intensity) across a 3.1 GHz frequency band. Finally, we study the dependence of the structure's performance on incident angle and slit width.

The ability to polarize and to manipulate the polarization state of electromagnetic radiation has been an essential tool ever since the wave nature of light was first understood. One such manipulation is the ability to rotate the polarization of linearly polarized radiation as it reflects or transmits through a structure. This is achieved via birefringence. Linear birefringence occurs in materials where orthogonal (*x* and *y*) components of electromagnetic radiation experience different refractive indices, in devices such as half- or quarter-waveplates that convert linearly polarized radiation into 90° rotated linear or circular polarizations respectively. Linearly birefringent structures possess anisotropy and hence their performance depends on their azimuthal orientation with respect to the incoming polarization. In contrast, circular birefringent materials possess different refractive indices for right (RCP) and left (LCP) circularly polarised radiation. This provides the potential to rotate linearly polarised radiation to any desired angle provided no circular dichroism, a differential absorption for LCP and RCP waves, is present.

Circular birefringence and dichroism are two manifestations of optical activity, which is exhibited by natural chiral media such as sugar solution. However, the degree of polarization rotation attainable through these structures is normally small and hence research in artificial chiral metamaterials^1^,^2^,^3^ (CMM), arrays of subwavelength chiral ‘meta-molecules', has received substantial interest. Collections of 3D (e.g. helical) elements have yielded circular polarizers and linear polarization rotators in transmission^4^,^5^,^6^. Planar 2D CMMs^7^, mounted onto a substrate, have been shown to rotate linear polarization^8^,^9^,^10^,^11^ despite having extremely subwavelength thicknesses. This rotation effect has been enhanced by using bilayers of chiral metamolecules^12^,^13^,^14^,^15^,^16^,^17^, layers of nonchiral meta-molecules rotated with respect to each other^18^,^19^,^20^,^21^,^22^,^23^, or via oblique incidence illumination of a nonchiral anisotropic planar metamaterial^24^.

Numerous structures have been used to achieve 90° polarization conversion via linear birefringence. Diffraction gratings are capable of linear conversion upon reflection^25^,^26^,^27^, and the bandwidth can be broadened by the excitation of bound surface modes^28^,^29^.This has been experimentally demonstrated in both the microwave^30^ and visible^31^,^32^,^33^,^34^ domains of the electromagnetic spectrum. More recently, the field of metamaterials has led to a similar result; polarization conversion upon reflection, using much thinner arrays of bianisotropic metallic elements above a ground plane. These geometries include elliptical patches in the visible regime^35^, dielectric cut-wire arrays^37^ and ‘L'-shaped holes^36^ in the infra-red, metallic cut-wires at terahertz frequencies^38^, and split-rings^39^,^40^ and ‘I' shaped wires^41^,^42^ in the microwave regime.

The underlying principle behind these structures, namely linear birefringence, is the same for the work we present in this study. However we demonstrate that a similar polarization conversion can be realised using a much simpler structure consisting of two orthogonal arrays of narrow slits in a metal sheet separated from a ground plane by a dielectric layer. This design would be beneficial when scaled to higher frequency regimes where fabrication is more difficult. Unlike relief gratings, polarization conversion from this array is a resonant effect associated with the dielectric cavity. Crucially, we use a very low-loss dielectric and optimise the geometry in order to minimize Joule heating resulting in less than 2% absorption. We design the structure so that the frequency of the cavity resonances results in a 3.1 GHz band across which 95% of incident radiation is converted. Finally, we study the effect of both incident angle and slit width on the polarization conversion and study the polarization state for all frequencies in the vicinity of the resonances.

The metamaterial studied consists of a perforated metallic layer separated from a ground plane by a dielectric. Initially, the upper surface contains a single set of continuous, sub-wavelength, parallel slits (referred to here as a monograting and shown schematically in Fig. 1a. Resonant microwave absorption occurs because electric field polarized parallel to the grating vector can diffract and excite lateral resonances within the dielectric cavities between adjacent slits^43^,^44^. At the fundamental resonant frequency the time-averaged electric field magnitude is maximum along the edge of the metallic strips due to a build up of charge. In the dielectric layer a standing wave is formed with field maxima at either side of the slit region and minima in the centre of each metallic strip and below the centre of the slit. This can be seen in Fig. 2 which shows, on resonance, the time-averaged electric field magnitude and the electric field vector of one unit cell of the structure across a plane perpendicular to the slit. Due to a rapid change of phase as the field diffracts through the slit, the structure resonates at a much lower frequency than expected due to the unit cell size, as explained in Ref. 44. Adding a second set of slits in the orthogonal direction, creating a bigrating (Fig. 1b), provides the structure with a second resonance. When the electric field is polarized at 45° to both sets of slits, the coupling efficiencies of the incident radiation to these resonances are equal. In this work we show how the cavity structure can be used as a polarization converting mirror. This is achieved by delaying the phase of one orthogonal component of the reflected electric field with respect to the other in much the same way as a half-wave plate. This phase delay is a frequency dependent quantity that is dictated by the cavity resonance(s).

Schematics of the two experimental structures studied are shown in Fig. 1. The samples have a dielectric thickness *t*_D_ = 0.787 mm and metal thickness *t*_M_ = 0.018 mm. This metal thickness is much greater than the penetration depth of copper at microwave frequencies and therefore the metal is considered to be a perfect electrical conductor. The upper layer of the monograting sample has a period of *λ*_g_ = 3.78 mm and slit width *w*_s_ = 0.125 mm and the dielectric, FR4, has the complex permittivity *ε* = 4.4 + *i*0.088. The bigrating sample has a short period in the *x*-direction of *λ*_gx_ = 3.2 mm and a long period in the *y* direction of *λ*_gy_ = 4.7 mm, with a slit width in both dimensions of *w*_s_ = 0.17 mm. The dielectric used is Rogers RT/Duroid 5880 with *ε* = 2.2 + *i*0.002. This material is chosen as it has a smaller imaginary part to its permittivity than FR4 and hence absorbs little power.

## Results and Discussion

Reflected intensity (reflectivity) spectra have been obtained for polarization angles of *ϕ* = 0°, 45°, 90°. For each of these incident polarizations both the polarization conserved and converted responses have been measured. In addition, co-polarized phase measurements are shown for *ϕ* = 0°, 90° as well as the ellipticity, γ, and handedness of the reflected radiation as a function of frequency. The experimental results are compared to finite element method modelling.

### Monograting

**Figure 3:**
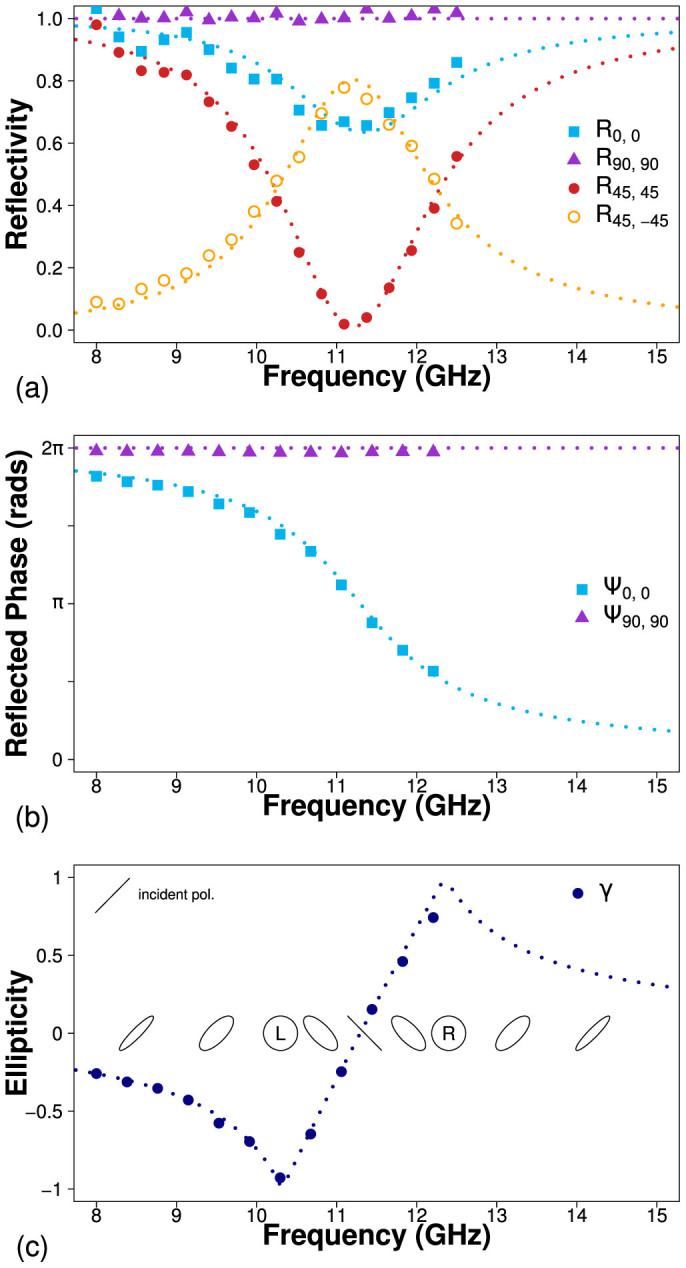
(a) Reflectivity as a function of frequency for the monograting sample. Co-polarized reflectivity for polarization angles of 0° (blue squares), 90° (purple triangles) and 45° (red circles) and cross-polarized reflectivity at *ϕ* = 45° (hollow yellow circles). (b) Relative phase of detected radiation as a function of frequency, normalized to the phase of reflection from a planar metallic mirror. (c) Ellipticity of reflected radiation, where negative and positive values indicate left and right handed respectively. Results from numerical modelling are shown as dotted lines.

Fig. 3a shows the normal-incidence reflectivity spectra for the monograting sample. The first subscript represents the polarization angle *ϕ* of the incident electric field and the second defines the respective orientation of the detecting antenna. The polarization converted reflectivity along the mirror planes of the sample (*R*_0,90_ and *R*_90,0_) have been omitted as there is no polarization conversion unless the symmetry of the surface is broken.

*R*_90,90_ shows that when the electric field is polarized parallel to the slits and along the *y*-direction, no radiation couples into the cavity. Instead, all radiation within the frequency range is reflected as if from a mirror and no features are present. This is supported by Fig. 3b which shows the phase of reflected radiation, normalized to that of a planar mirror. Ψ_90,90_ shows no difference between the responses from the structure and the mirror and it reflects with a phase change *δ*Ψ_y_ = *π* at all frequencies within the range.

The co-polarized reflectivity when the electric field is polarized perpendicular to the slits (*x*-direction), *R*_0,0_, shows a minimum at 11.2 GHz as the electric field diffracts through the slits and excites a lateral Fabry-Perot-like resonance in the dielectric cavity beneath, where ~35% of the incident power is absorbed. The corresponding phase measurement displays the characteristic change in phase through the resonance when normalized to the reflected phase of a planar mirror. At the resonant frequency of 11.2 GHz, the phase of reflection from the monograting Ψ_0,0_ is *π* out of phase with that from the mirror, indicating that at this frequency, the total change in phase upon reflection is *δ*Ψ_x_ = 0, 2*π*. Away from the resonance, beyond the limits of the figure, the phase of reflected radiation returns to that of the mirror hence *δ*Ψ_x_ = ±*π*.

When the incident radiation is polarized at *ϕ* = 45° to the slits, the electric field can be decomposed into equal components in the *x* and *y* directions such that, assuming no losses,
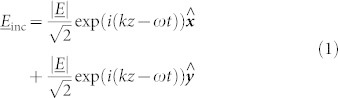

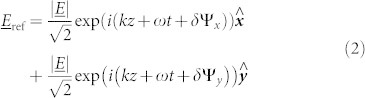


**Figure 4:**
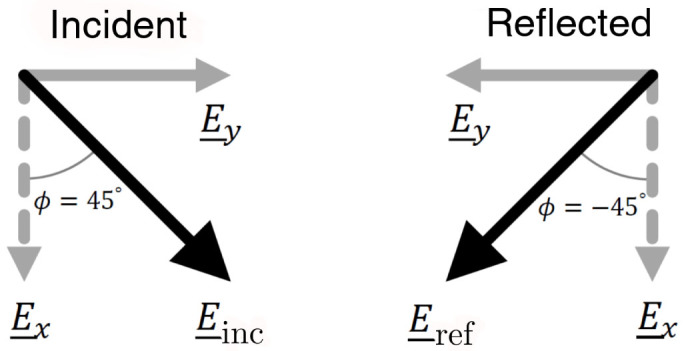
Schematic diagram showing the incident and reflected polarization states at the resonant frequency of 11.2 GHz when the electric field is polarized at 45° to the grating slits.

Hence, at 11.2 GHz, half of the incident power is reflected from the upper surface with a phase change *δ*Ψ_y_ = *π* and the other half diffracts into the resonant cavity mode which subsequently reradiates with a *δ*Ψ*_x_* = 2*π* phase change. Due to the phase difference *δ*Ψ_x_ − *δ*Ψ_y_ = *π* between these processes, the combined reflected field is polarized perpendicular to the incident field as portrayed in Fig. 4. The result of this is that a peak is observed in the cross-polarized reflectivity signal *R*_45,-45_. This power is no longer detected in the co-polarized state and hence a minimum is observed in *R*_45,45_. Due to loss in the dielectric, the peak in polarization conversion cannot reach 100%. Selecting a dielectric material with a smaller imaginary part of its permittivity would minimize loss and maximize the polarization conversion, hence the switch from FR4 to Rogers RT/Duroid 5880 for the bigrating sample discussed in the next section. The phase difference *δ*Ψ_x_ − *δ*Ψ_y_ is frequency dependent and away from the resonance the reflected polarization state is elliptically polarized. Fig. 3c shows the ellipticity, *γ*, of the reflected wave as a function of frequency, where the ellipticity is defined as the ratio of the semi-minor to semi-major axis of the polarization ellipse. Positive and negative values indicate right and left handed polarization respectively. Hence, at 10.3 GHz and 12.4 GHz, where *δ*Ψ_x_ − *δ*Ψ_y_ = 3*π*/2, *π*/2, the reflected radiation is circularly polarized, with left and right handedness respectively. This polarization state assumes a linearly polarized incident beam with *ϕ* = 45°. An incident polarization of *ϕ* = −45° would cause the reflection to have the opposite handedness.

The response of the structure to circularly polarized incident light can be determined by decomposing the electric field into the *x* and *y* directions. We can therefore infer the circular polarization conversion (not shown) from the reflected amplitude and phase of linear polarization. At the resonant frequency of the structure, circularly polarized light is reflected with the opposite handedness, where the handedness is defined in relation to the direction of propagation. Furthermore, at 10.3 GHz and 12.4 GHz, where *δ*Ψ_x_ − *δ*Ψ_y_ = 3*π*/2, *π*/2, the structure now acts as a circular to linear converter.

### Bigrating

**Figure 5:**
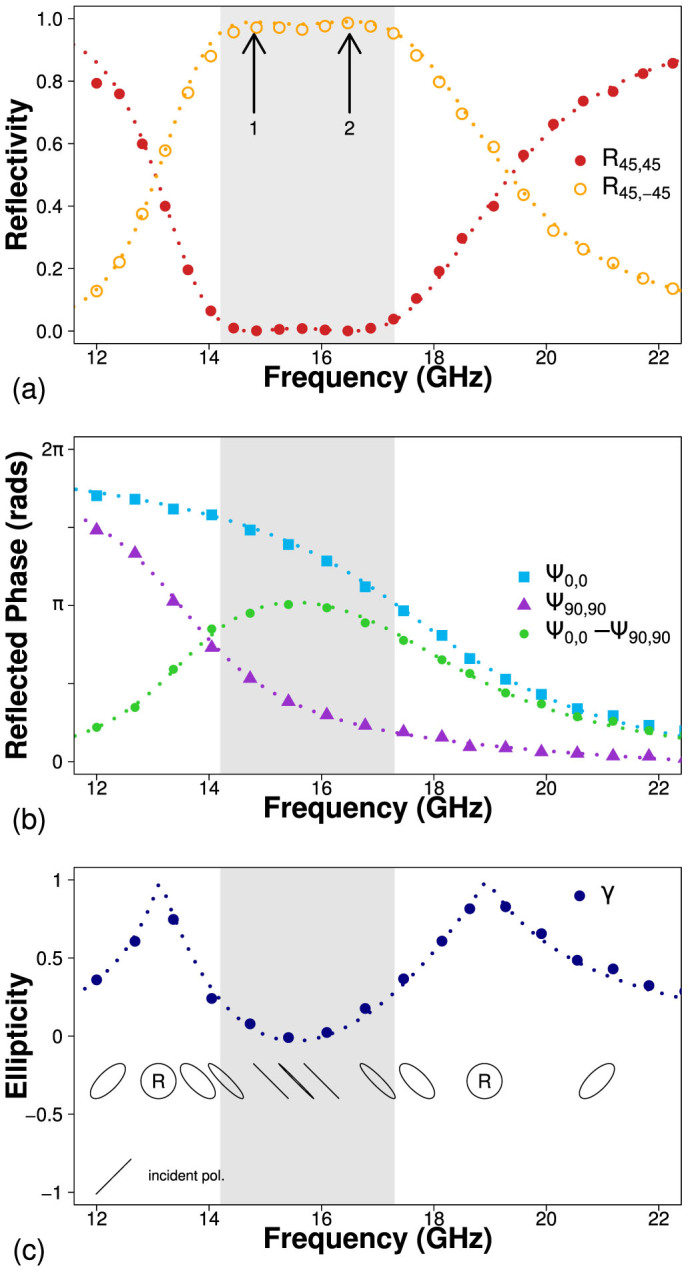
(a) Co-polarized (filled red circles) and cross-polarized (hollow yellow circles) reflectivity for *ϕ* = 45°. (b) Relative phase of co-polarized detected radiation as a function of frequency, normalized to the phase of reflection from a planar metallic mirror, for 0° (blue squares), 90° (purple triangles) and their difference (green circles) (c) Ellipticity of reflected radiation, where negative and positive values indicate left and right handed respectively. Results from numerical modelling are shown as dotted lines.

Now we consider the bigrating cavity array in order to broaden the frequency range over which polarization conversion can be achieved. Fig. 5a shows the reflectivity as a function of frequency for the bigrating structure. The upper metal surface now contains periodic slits in both the *x* and *y* directions and hence cavity resonances can be excited in both directions. It has been shown that when the pitches of these perpendicular slits are equal, *λ*_gx_ = *λ*_gy_, a polarization independent cavity resonance exists and there is no polarization conversion due to the four-fold rotational symmetry of the structure^45^. If the pitches are not equal, the two orthogonal resonances separate in frequency and polarization conversion is possible provided the electric field is not polarized along a mirror plane of the surface. The bigrating sample is designed to minimize absorption in the dielectric cavity such that the resonant dips in reflectivity cannot be clearly resolved. Hence, the reflectivity data *R*_0,0_ and *R*_90,90_ are omitted although the phase data in Fig. 5b shows two resonant features centred at 13.5 GHz and 17.4 GHz.

When the incident electric field is polarized at 45° to both sets of slits, two peaks in polarization conversion arise, labelled with arrows in Fig. 5a. For this particular geometry, the two resonance frequencies are sufficiently separated such that the minimum between the two peaks is minimized in *R*_45,-45_. There exists a 2.5 GHz frequency band where 98% of incident radiation is reflected with the othogonal polarization and the remaining 2% is absorbed. This bandwidth increases to 3.1 GHz over which 95% of the radiation is converted, indicated by the gray region in Fig. 5. The phase and ellipticity data presented in Fig. 5b–c shows that the phase difference between the *x* and *y* components of the reflected electric field, Ψ_0,0_ − Ψ_90,90_, is approximately equal to *π* over the same frequency range, causing linear polarization conversion. As before, circularly polarized radiation would be reflected from this structure with the opposite handedness, where the handedness is defined in relation to the direction of propagation.

### Slit width

**Figure 6:**
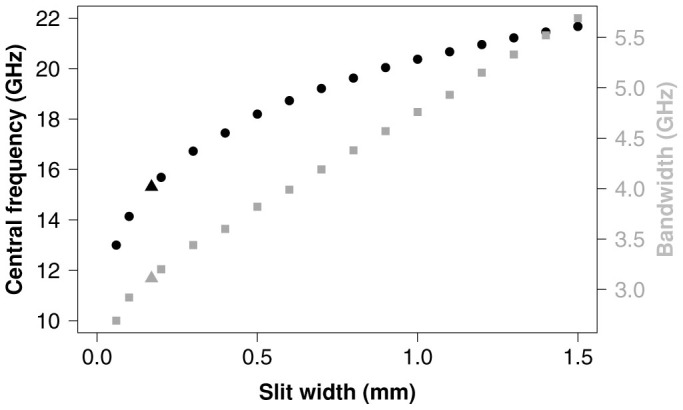
The bandwidth (grey squares) and central frequency (black circles) of the 95% polarization converting band for various slit widths *w*_s_. The values corresponding to the bigrating sample are shown as triangles.

Fig. 6 shows how the bandwidth and central frequency of the polarization converting band varies with slit width *w*_s_ according to numerical modelling. The data points corresponding to the sample studied thus far are shown as triangles. The resonant frequency of each cavity depends not just on cavity volume but also on this separation between cavities. This is because a narrower slit results in a steeper field gradient beneath the slit as the incident field diffracts. The field undergoes a *π*/2 phase change over a distance of the order of the slit width which leads to an effective lengthening of the cavity^44^. The central frequency data (black circles) shown in Fig. 6 show how the polarization conversion band decreases in frequency as the slit is narrowed. This trend is very similar to that reported in Ref. 44 for the transmission resonance of a structured slit. The 95% conversion bandwidth (grey squares) also decreases with decreasing slit width. The range of slit widths between 0.06 and 1.5 mm were chosen as outside of this range the conversion is reduced below 95% at the central frequency. This data shows how the central frequency and the bandwidth of operation can be tailored by making a small variation to the design of the bigrating.

### Incident angle

**Figure 7:**
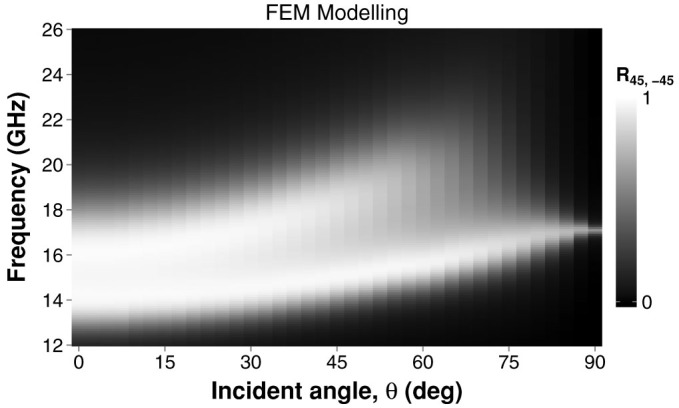
Cross-polarized reflectivity of the bigrating sample with incident polarization angle *ϕ* = 45°, as a function of both frequency and incident angle *θ*.

In Fig. 7 the numerically modelled cross-polarized reflectivity is presented as a function of frequency and incident angle *θ*. The 95% conversion band remains up to an angle of 30°, where the bandwidth has increased to 3.55 GHz whilst the central frequency has shifted up to 15.9 GHz from 15.3 GHz. Beyond 30°, the amplitudes of both conversion peaks reduces and they move further up in frequency.

### Linear to circular conversion

**Figure 8:**
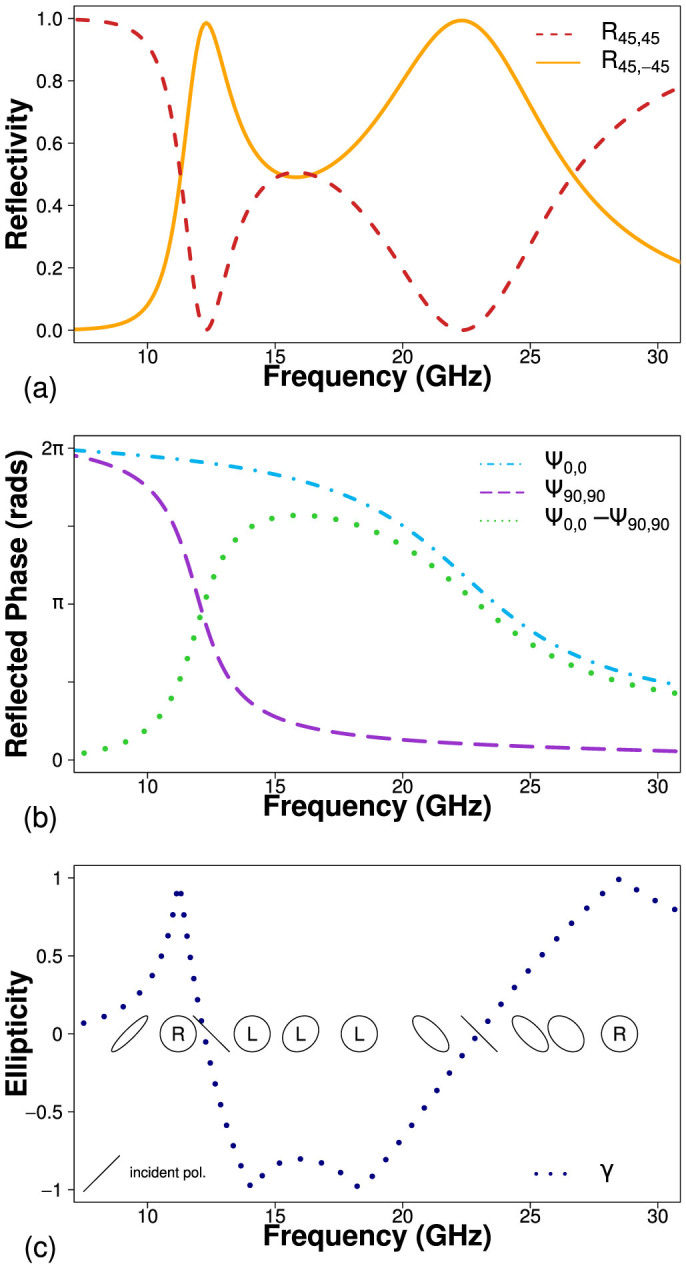
Numerical modelling results for the linear to circular polarization converting bigrating structure. (a) Co-polarized (dashed red line) and cross-polarized (solid yellow line) reflectivity for *ϕ* = 45°. (b) Relative phase of reflected radiation as a function of frequency, normalized to the phase of reflection from a planar metallic mirror, along the *x* direction (dot-dash), *y* direction (dashed) and their difference (dotted). (c) Ellipticity of reflected radiation, where negative and positive values indicate left and right handed respectively.

Just as this bigrating structure acts as a reflecting half wave plate, it is also possible to achieve a reflecting quarter wave plate (to convert between linear and circular polarization) by separating the two orthogonal resonances further. Numerical modelling for the bigrating array but now with a short period of *λ*_gx_ = 5.4 mm and a long period of *λ*_gy_ = 2.05 mm is shown in Fig. 8. Between 14.5 and 17 GHz, both the co-polarized, *R*_45,45_, and the cross-polarized, *R*_45,-45_, reflectivity is ~0.5, hence the reflected signal must be circularly polarized. This is verified by the phase and ellipticity data in Fig. 8b–c. Ψ_0,0_ − Ψ_90,90_ shows that the difference in phase between the reflected *x* and *y* components of the electric field is equal to 3*π*/2.

## Conclusion

In this paper, a metamaterial consisting of a grating separated from a ground plane by a low-loss dielectric has been experimentally optimised for polarization conversion in reflection. The result is a 95% polarization converting mirror for normally incident microwave radiation with its electric field polarized at 45° to the two orthogonal gratings. Using a rectangular bigrating as the upper surface of the array allows an increase of the operating bandwidth to 3.1 GHz centred at 15.3 GHz. This structure benefits from an uncomplicated geometry which could be scaled to higher frequency regimes. It is very thin (*λ*/25) and the unit cell size is also subwavelength (*λ*/4). Most importantly, the absorption is very small (2%) meaning less incident power is lost upon reflection than previous designs^35^,^36^,^37^,^38^,^39^,^41^,^42^. Numerical modelling has shown that the polarization conversion performance is maintained for incident angles up to 30° and that the central frequency and bandwidth of operation can be varied by changing the slit width. Further modelling shows that by changing the dimensions of the cavity array it can also function as a linear to circular polarization converter across the same frequency range.

## Methods

### Fabrication

Both the monograting and the bigrating samples were produced using standard lithographic etching of a 400 mm × 600 mm double-sided printed circuit board.

### Experiment

Microwave radiation is emitted from a narrow-band horn antenna and is collimated using a parabolic mirror into a ~ 300 mm diameter beam which illuminates the sample with an approximate plane wave at close to normal incidence (*θ* < 3°). The same mirror refocuses the reflected beam to the detecting horn placed adjacent to the source. The sample itself is rotated to vary the polarization angle, *ϕ*, and the detector is rotated with respect to the source to measure either the co-polarized or cross-polarized signal.

### Modelling

Finite element method modelling was performed using Ansoft HFSS. Master/slave boundary conditions were used to reduce the dimensionality of the structure to that of a single unit cell and a Floquet Port excitation was placed 12 mm above the upper metal surface. A solution was obtained for a frequency of 30 GHz and a frequency sweep used to produce the results shown in this paper.

## Author Contributions

B.T. obtained experimental data and conducted numerical modelling. H.J.R. initiated sample fabrication and assisted with experimental techniques. A.P.H. and J.R.S. directed the research. All authors helped to analyse the results and contributed to the manuscript.

## Figures and Tables

**Figure 1:**
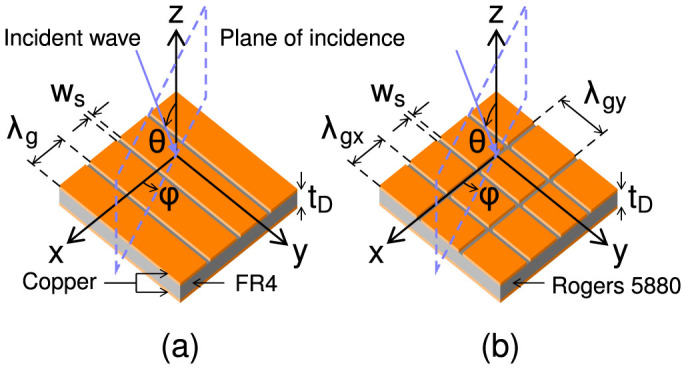
Sample geometry and coordinate system for (a) the monograting cavity array with period *λ*_g_ and slit width *w*_s_ and (b) the bigrating cavity array with periodicity *λ*_gx_ and *λ*_gy_ in the *x* and *y* directions respectively.

**Figure 2:**
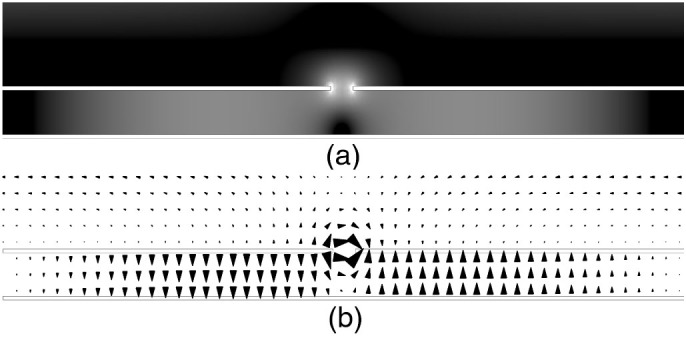
A cross-section of a single unit cell of a typical monograting structure perpendicular to the slit at the fundamental resonant frequency. The metallic sections are shown as bordered white regions. (a) Resonant time-averaged electric field magnitude where black to white represents increasing field strength. (b) Resonant electric field vector at a phase corresponding to maximum field enhancement, showing both the quantisation and reversal of the field beneath the metallic strip.
